# Characterization, Expression, and Functional Analysis of a Novel NAC Gene Associated with Resistance to Verticillium Wilt and Abiotic Stress in Cotton

**DOI:** 10.1534/g3.116.034512

**Published:** 2016-10-24

**Authors:** Weina Wang, Youlu Yuan, Can Yang, Shuaipeng Geng, Quan Sun, Lu Long, Chaowei Cai, Zongyan Chu, Xin Liu, Guanghao Wang, Xiongming Du, Chen Miao, Xiao Zhang, Yingfan Cai

**Affiliations:** * State Key Laboratory of Cotton Biology, College of Life Science, Henan Key Laboratory of Plant Stress Biology, Henan University, Kaifeng, 475004 Henan, China; † College of Bioinformation, Chongqing University of Posts and Telecommunications, 400065 Sichuan, China; ‡ State Key Laboratory of Cotton Biology, Cotton Institute of the Chinese Academy of Agricultural Sciences, Key Laboratory of Cotton Genetic Improvement, Ministry of Agriculture, Anyang, 455000 Henan, China; § Kaifeng Academy of Agriculture and Forestry, 475004 Henan, China

**Keywords:** NAC transcription factor, Verticillium wilt, VIGS, biotic and abiotic stress, cotton

## Abstract

Elucidating the mechanism of resistance to biotic and abiotic stress is of great importance in cotton. In this study, a gene containing the NAC domain, designated *GbNAC1*, was identified from *Gossypium barbadense* L. Homologous sequence alignment indicated that *GbNAC1* belongs to the TERN subgroup. *GbNAC1* protein localized to the cell nucleus. *GbNAC1* was expressed in roots, stems, and leaves, and was especially highly expressed in vascular bundles. Functional analysis showed that cotton resistance to Verticillium wilt was reduced when the *GbNAC1* gene was silenced using the virus-induced gene silencing (VIGS) method. *GbNAC1*-overexpressing *Arabidopsis* showed enhanced resistance to *Verticillium dahliae* compared to wild-type. Thus, *GbNAC1* is involved in the positive regulation of resistance to Verticillium wilt. In addition, analysis of *GbNAC1*-overexpressing *Arabidopsis* under different stress treatments indicated that it is involved in plant growth, development, and response to various abiotic stresses (ABA, mannitol, and NaCl). This suggests that *GbNAC1* plays an important role in resistance to biotic and abiotic stresses in cotton. This study provides a foundation for further study of the function of NAC genes in cotton and other plants.

Cotton is an important economic crop, but it is susceptible to Verticillium wilt, a soil-borne vascular disease that can result in devastating losses of yield and quality; the leaves turn yellow, the plant defoliates, and even dies when infected by *V. dahliae* (Sink and Grey 1999). Currently, the molecular mechanisms of resistance to Verticillium wilt remain unclear.

NAC protein is involved in various biotic (pathogen attack) and abiotic stress responses (salinity, temperature, and drought), as well as in developmental processes including cell division (Kim *et al.* 2006), embryo development (Duval *et al.* 2002), leaf senescence (Breeze *et al.* 2011), vascular vessels (Yamaguchi *et al.* 2010), seed development (Sperotto *et al.* 2009), lateral root development (Xie *et al.* 2000), fiber development (Ko *et al.* 2007), and shoot apical meristem formation (Kim *et al.* 2007). The cotton stress-responsive NAC transcription factor also plays important roles in the stress response to both abiotic and biotic stresses by coordinating phytohormone signaling networks (He *et al.* 2016).

The NAC protein family is a large group of plant transcription factors and its name is derived from the three initially discovered genes: NAM (no apical meristem), ATAF1/2, and CUC2 (cup-shaped cotyledon) (Duval *et al.* 2002; Grant *et al.* 2010; Ooka *et al.* 2003; Su *et al.* 2013). There are more than 117 NAC genes in *Arabidopsis* (Nuruzzaman *et al.* 2010), 151 in *Oryza sativa* (Nuruzzaman *et al.* 2010), 154 in *G. raimondii* (Shang *et al.* 2013), and 60 full-length putative NAC genes in *G. hirsutum* L (Syed *et al.* 2014). NAC proteins contain a highly conserved NAC domain, consisting of about 150 amino acids in the N-terminus, and a divergent transcriptional regulatory domain in the C-terminus (Aida *et al.* 1997; Ooka *et al.* 2003). Based on an analysis of the structure of the NAC domain in rice and *Arabidopsis*, NAC proteins were divided into two large groups (I and II). There are 14 subgroups (TERN, ONAC022, and SENU5, etc.) in group I and four subgroups (ANAC001, ONAC003, ONAC001, and ANAC063) in group II (Ooka *et al.* 2003). The NAC domain contains five subdomains (A–E), and it plays an important role in DNA binding, formation of homodimers or heterodimers, and nuclear localization (Olsen *et al.* 2005).

A recent study found that the overexpression of a rice NAC gene (*SNAC1*) could improve drought and salt tolerance in transgenic cotton (Liu *et al.* 2014b). *AtLOV1*, an *Arabidopsis* NAC transcriptional factor, regulates the cold response and flowering time (Yoo *et al.* 2007). *AhNAC2*-overexpressing tobacco plants had improved salt tolerance (Liu *et al.* 2011). *NTL8*, an *Arabidopsis* NAC protein, regulates GA3-mediated salt signaling in seed germination (Kim *et al.* 2008). *ANAC019* and *ANAC055* are transcription activators regulating the JA-induced expression of defense genes (Bu *et al.* 2008). The *SiNAC* gene is involved in stress and developmental regulation in plants (Puranik *et al.* 2011). In *Arabidopsis*, some NAC related proteins (*NST1*, *NST2*, and *NST3*) were shown to regulate secondary cell wall biosynthesis (Mitsuda *et al.* 2005, 2007; Mitsuda and Ohme-Takagi 2008). *GhXND1*, a NAC transcription factor in *G. hirsutum*, may be related to the regulation of plant xylem development (Li *et al.* 2014).

Studies suggest that the NAC proteins play a pivotal role in the plant innate immune system, in terms of both systemic acquired resistance and basic defense. Grapevine *NAC1* is involved in the regulation of the disease resistance response (Le Hénanff *et al.* 2013). Rice NAC genes (*OsNAC6*, *ONAC122*, *RIM1*, and *ONAC131*) were reported to have a regulatory function in disease resistance against *Magnaporthe oryzae* and rice dwarf virus (Nakashima *et al.* 2007; Motoyasu *et al.* 2009; Yoshii *et al.* 2010; Sun *et al.* 2013a). Recent research demonstrated that *VpNAC1* may function as a positive regulator in resistance to *Erysiphe cichoracearum* and *Phytophthora parasitica* var. *nicotianae* Tucker (Zhu *et al.* 2012). *HvNAC6* in barley can improve resistance against virulent *Blumeria graminis f. sp. hordei* and enhance penetration resistance (Chen *et al.* 2013; Jensen *et al.* 2007).

In this study, we identified the *GbNAC1* gene from previous transcriptome sequencing of *G. barbadense*. The function of *GbNAC1* was analyzed using VIGS and overexpression approaches. Results revealed that *GbNAC1* plays important roles in resistance to Verticillium wilt and abiotic stress in cotton.

## Materials and Methods

### Materials and growth conditions

Cotton varieties Xinhai 15 and Xinhai 16 (*G. barbadense* L.) were cultivated in a constant temperature incubator under long day conditions (16 hr light/8 hr dark) with about 80% relative humidity at 28/25° (day/night). *Arabidopsis thaliana* (ecotype Columbia-0) was grown in a 16 hr light/8 hr dark photoperiod with about 125 μE m^−2^s^−1^ light at 23°. *Nicotiana benthamiana* was grown in an incubator maintained at 25° with a 14 hr light/10 hr dark photoperiod for use after 3 wk. *V. dahliae* strain V991 (highly aggressive) was cultivated on potato dextrose agar (PDA) at 25° for 1 wk, and was then inoculated into Czapek medium until the concentration of conidia was 10^7^/ml for use.

### Preparation of cDNA and gDNA

Total RNA was extracted from whole Xinhai 15 (*G. barbadense L*.) plants using the Plant RNA EASYspin Plus Kit (Aidlab, Beijing, China), according to the manufacturer’s instructions. First strand cDNA was synthesized using the Superscript II RNase H-Reverse Transcriptase Kit (Takara Biotechnology, Dalian, China). Genomic DNA was isolated from young cotton leaves using the Plant Genomic DNA Kit (Tian Gen, Beijing, China).

### Cloning of GbNAC1 and phylogenetic trees

Using a unigene fragment (unigene25035_kv-1) and *in silico* cloning, the cDNA sequence of *GbNAC1* was obtained. Primers (*GbNAC1*F: 5′-ATGGTTGCAGAGCTTGCTGG-3′; *GbNAC1*R: 5′-TTATATTTCATTAATTTGTC-3′) were designed to amplify the cDNA template from Xinhai 15 and this sequence was verified. The open reading frame (ORF) was predicted by ORF Finder (https://www.ncbi.nlm.nih.gov/orffinder/). Multi-sequence alignments were obtained using DNAMAN software. MEGA software was used to construct phylogenetic trees with the neighbor-joining (NJ) method.

Putative NAC-TF genes were identified from protein data of *G. raimondii* UIbr, which was downloaded from the Cotton Genome Project (http://cgp.genomics.org.cn/) by HMMER. The NJ phylogenetic tree generated for the ORF region of NAC-TF genes and motifs was annotated using MEME (http://meme-suite.org/tools/meme) (Bailey and Elkan 1994).

### Quantitative RT-PCR analysis

Cotton RNA was extracted from roots, stems, euphyllas, cotyledons, and stem apices from Xinhai 15 and Xinhai 16 at the two-true-leaf growth stage. Each Xinhai 15 seedling was inoculated with *V. dahliae* spore suspension (2 × 10^7^ spores/ml) through injured roots, while control seedlings were treated with distilled water in the same way. Euphyllas were collected from three repeats after treatment for 0, 1, 4, 8, 12, 24, 36, 48, and 72 hr. RNA was isolated from leaves and qRT-PCR was performed.

qRT-PCR was performed with three replicates using an ABI 7300 Real Time PCR system and SYBR Premix Ex *Taq* (Takara). The cotton ubiquitin gene (GbUBQ7F: 5′-GAAGGCATTCCACCTGACCAAC-3′; GbUBQ7R: 5′- CTTGACCTTCTTCTTCTTGTGCTTG-3′) was used as a standard control. Gene-specific primers (qGbNAC1F: 5′-GACCTTGAGCCTTGGGACC-3′; qGbNAC1R: 5′-CTTCCCTCTCTTGTCTTGGTGTA-3′) were used for amplification.

### Subcellular localization

The GbNAC1 protein was amplified using the nlsGbNAC1F (5′-AAAAAGCAGGCT ATGGTTGCAGAGCTTGCTGG-3′, attB1 adaptor sequence underlined) and nlsGbNAC1R (5′-AGAAAGCTGGGTC TATTTCATTAATTTGTC-3′, attB2 adaptor sequence underlined) primers. The PCR product was cloned into the pK7FWG2.0 vector using the BP and LR reactions. The constructs were cloned into *Agrobacterium tumefaciens* strain GV3101, while empty pK7FWG2.0 was used as a control. The agrobacterial culture suspensions for both control and constructs were injected into the underside of *N. benthamiana* leaves cultured for 3 wk using a 20 ml needleless syringe. After dark incubation of the injected tobacco for 48 hr, the injected leaves were observed with a confocal laser scanning microscope (Zeiss LSM710; Carl Zeiss, Oberkochen, Germany). Then, 4, 6-diamino-2-phenyl indole (DAPI) was used to stain cell nuclei.

### Promoter analysis

The promoter sequence was amplified from gDNA using the pGbNAC1F (5′-AAAAAGCAGGCTGACTTGTAAACTGGTGCCTAT-3′, attB1 adaptor sequence underlined) and pGbNAC1R (5′-AGAAAGCTGGGTGTAGCTGATCTATACGTGTTGT-3′, attB1 adaptor sequence underlined) primers. The PCR product was cloned into the pKGWFS7.0 vector using the BP and LR reactions. The constructed plasmid was cloned into *Ag. tumefaciens* strain GV3101. Transformation of *Arabidopsis* plants was performed using the floral dip method. For selection, seeds were planted in aseptic conditions on MS agar containing 25 mg/L kanamycin. The selected positive seedlings were histochemically stained for GUS activity based on the method of Jefferson (1987).

### Construction of the VIGS vector and transient expression

A 266 bp gene-specific fragment from *GbNAC1* was PCR amplified using vGbNAC1F (5′-CCGGAATTCTCATACTTGTAGACCAAAGGAAC-3′, *Eco*RI restriction site underlined) and vGbNAC1R (5′-CGGGGTACCAGTGATAGCATAGAAAAGCAATA-3′, *Kpn*I restriction site underlined) primers. The PCR product was cloned into the pTRV2 vector to produce TRV:GbNAC1. Then the recombinant plasmids pTRV1, pTRV2, and TRV:GbNAC1 were transformed into *Ag. tumefaciens* strain GV3101.

The VIGS transient expression methods followed Gao *et al.* (2011). VIGS-infiltrated seedlings were allowed to grow for 2 wk until the two-true-leaf stage and then leaves were collected and stored at −80°. Seedlings were simultaneously inoculated with a *V. dahliae* spore suspension (2 × 10^7^ spores/ml) through injured roots and disease progression was analyzed.

### Construction of the plant overexpression vector and generation of transgenic plants

The ORF of *GbNAC1* was amplified from cDNA with *oeGbNAC1*F (5′-GCTCTAGAATGGTTGCAGAGCTTGCTGG-3′, *Xba*I restriction site underlined) and *oeGbNAC1*R (5′-GGACTAGTTTATATTTCATTAATTTGTCCCCA-3′, *Spe*I restriction site underlined) primers. The PCR fragment was cloned into the super-pCAMBIA1300 vector. The super-pCAMBIA1300:*GbNAC1* construct was transformed into *Ag. tumefaciens* GV3101. Transformation of *Arabidopsis* plants was performed using the floral dip method. For selection, seeds were planted in aseptic conditions on MS agar containing 25 mg/L hygromycin for three generations. T3 lines displaying 100% hygromycin resistance were considered homozygous, and were used to observe the phenotype of transgenic plants and for further experiments.

### Overexpression of GbNAC1 in Arabidopsis and responses to abiotic and biotic stresses

To further explore the function of *GbNAC1*, homozygous seeds from T3 lines of overexpressing transgenic plants were used for abiotic stress treatment. To observe germination and plant growth, seeds from wild-type and transgenic plants were sown in triplicate on blank MS medium and MS medium with 120 mM NaCl, 240 mM mannitol, or 1 μM ABA. Seeds were incubated for 2–3 d at 4° in darkness to break dormancy, and then were transferred to the culture room (21°). Germination was recorded daily for 5 d, when the radicle emerged. Wild-type and transgenic plants were photographed after 10 d.

Overexpressing transgenic plants were also used to study the function of *GbNAC1* in response to *V. dahliae*. Wild-type and transgenic *Arabidopsis* plants were cultivated in a growth chamber (according to the above-mentioned growth conditions). After 25 d, plants were gently removed from the vermiculite and the roots were washed with sterile water. Then, the roots were dipped in a fresh *V. dahliae* spore suspension of 10^7^ spores/ml. The seedlings were replanted in pathogen-free vermiculite after inoculation and cultivated under normal conditions. Seedling growth was monitored until disease symptoms appeared.

### Data availability

The authors state that all data necessary for confirming the conclusions presented in the article are represented fully within the article.

## Results

### Characterization of the GbNAC1 gene

A 1159 bp unigene containing a NAM domain was obtained through screening differentially expressed genes involved in the cotton defense response to *V. dahliae* (Sun *et al.* 2013b). The assembled sequence obtained after *in silico* cloning was the same as the original unigene sequence. Through T-vector cloning and sequencing, a verified ORF of 543 bp (GenBank ID: KP317496) encoding a protein of 180 amino acids was obtained using ORF Finder.

**Figure 1:**
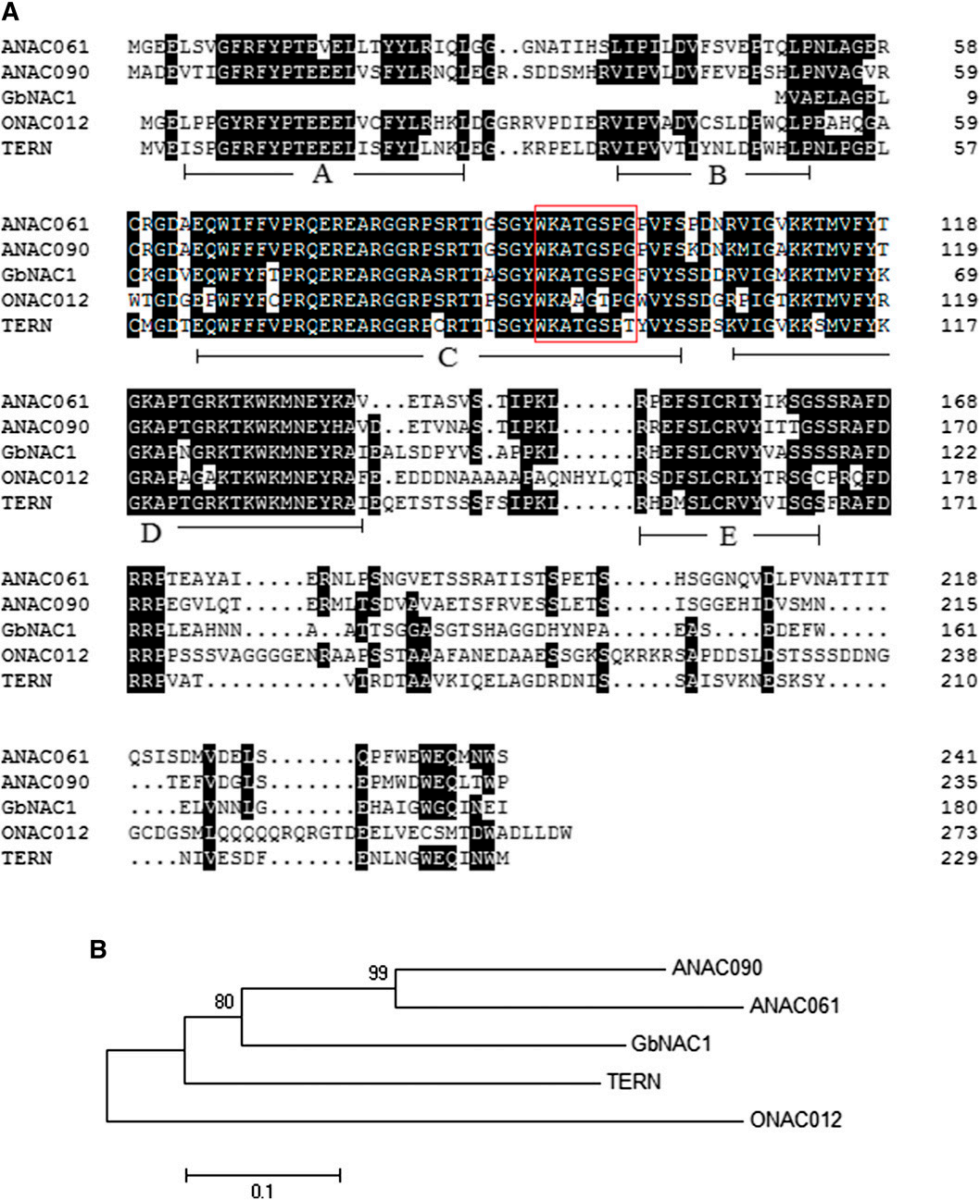
Characterization of *GbNAC1*. (A) Amino acid sequence alignment of *GbNAC1* with other TERN group NAC proteins by DNAMAN software. Identical amino acids are highlighted in black and the conservative domain is boxed in red. The proteins involved are *ANAC061* (NP_001118771.1) and *ANAC090* (NP_197630.1) from *Arabidopsis*, *TERN* (XP_009759500.1) from *N. tabacum*, and *ONAC012* (NP_001055672.1) from *O. sativa*. The location of five conserved substructural domains (A–E) is shown below the sequences. (B) Phylogenetic relationship of above NAC proteins. Bootstrap values were 500 replicates with neighbor-joining method using MEGA 5.0.

*GbNAC1* belongs to the TERN subgroup of the NAC-TF family, according to the multiple alignment (Figure 1A) and phylogenetic tree (Figure 1B). The typical NAC domain, located at the N-terminus, contains five subdomains (A–E), but *GbNAC1* lacks subdomains A and B. NAC proteins also have a conserved sequence, WKATGSPG.

### Phylogenetic tree and conserved motif analysis of GbNAC1 and G. raimondii NAC-TF genes

**Figure 2:**
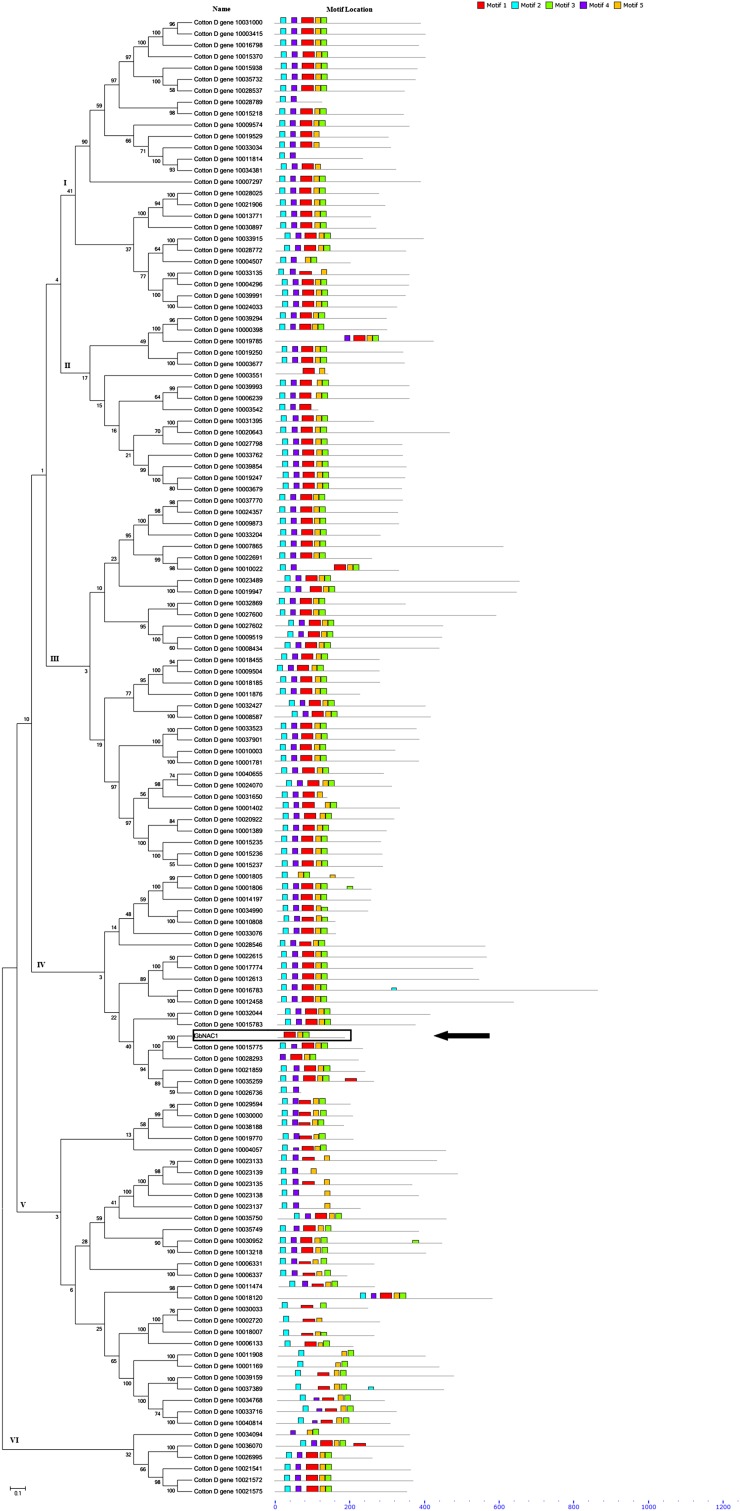
Phylogenetic relationships and conserved motifs of *GbNAC1* and the other NAC genes in *G. raimondii*. The phylogenetic tree was constructed using MEGA 5.0 by NJ method with 500 bootstrap replicates on a multiple alignment of 129 amino acid sequences of NAC genes from *G. raimondii* and *GbNAC1*. Schematic diagram of the conserved motifs were showed by MEME. Each motif is represented by a colored box and the black lines represent the nonconserved sequences. *GbNAC1* is marked by a black box and a black arrow.

To determine the location of *GbNAC1* in cotton, a phylogenetic tree of 129 NAC-TF genes from *G. raimondii* using HMMER 3.0 software (E-value < e^−10^ and deleting those proteins without a NAC domain) was constructed. The result indicates that *GbNAC1* is close to the Cotton D gene 10015775 (Figure 2). The MEME program was used to predict putative conserved motifs, and five motifs were found at the N-terminus corresponding to the five NAC subdomains. As expected, *GbNAC1* lacks the first and the second motif, namely the A and B subdomains. The phylogenetic tree shows six distinct subfamilies (I, II, III, IV, V, and VI) and each subfamily was consistent with similar conserved motifs.

### Subcellular localization of GbNAC1

**Figure 3:**
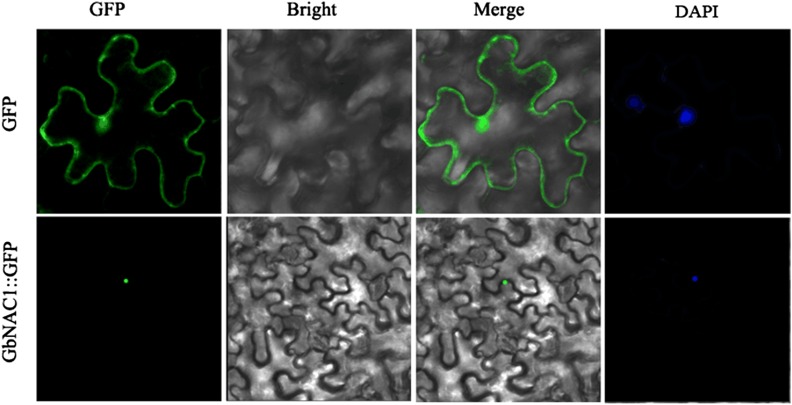
Subcellular localization of *GbNAC*1 in a tobacco epidermal cell visualized by Zeiss LSM710.

To determine the intracellular localization of *GbNAC1*, the *GbNAC1*::GFP fusion gene was constructed and transferred into *N. benthamiana* leaves. As shown in Figure 3, the fusion protein targeted to the nucleus of transgenic tobacco epidermal cells and the DAPI staining verified this result, indicating that the *GbNAC1* protein is localized in the cell nucleus.

### Expression pattern of GbNAC1 in cotton tissues and induction by V. dahliae

**Figure 4:**
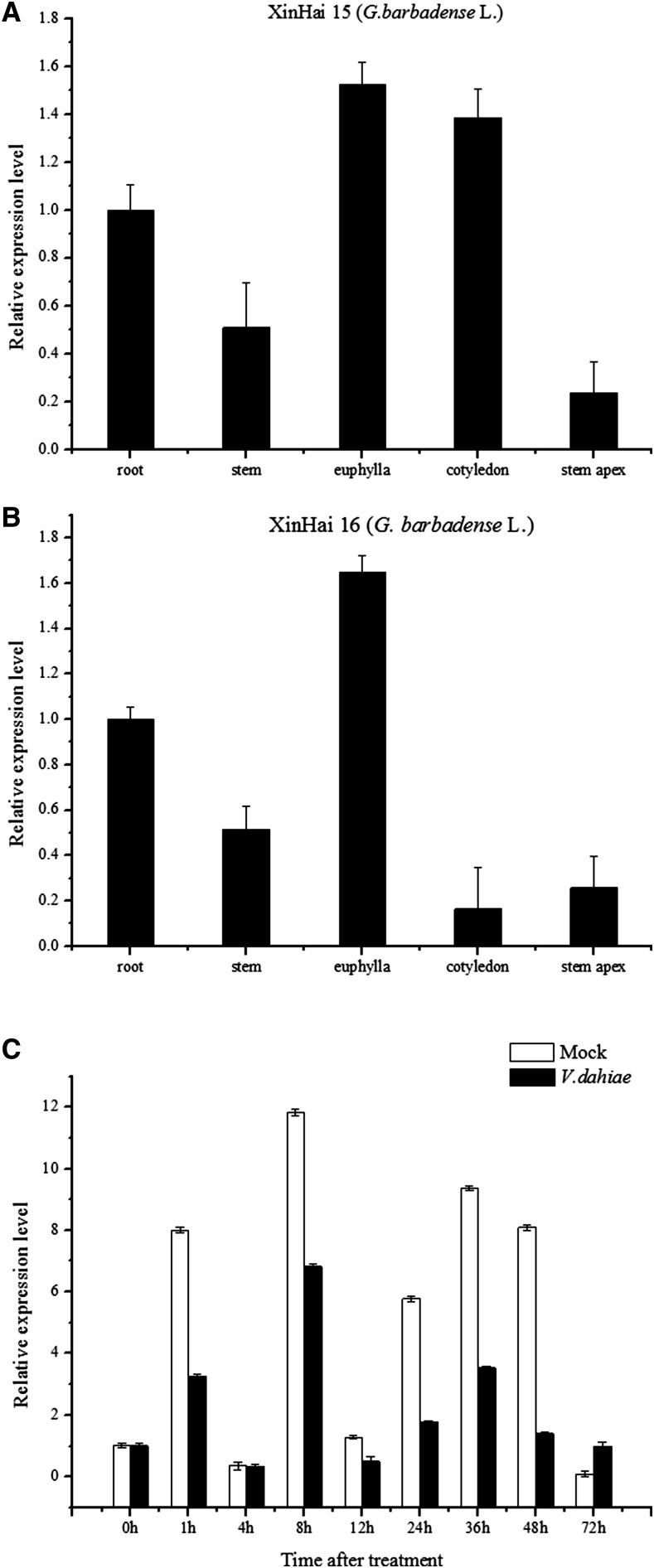
qRT-PCR analysis of expression profile of *GbNAC1*. (A) Tissue-specific expression pattern of *GbNAC1* in Xinhai 15. (B) Tissue-specific expression pattern of *GbNAC1* in Xinhai 16. (C) Expression of *GbNAC1* in response to *V. dahliae*. The leaves were collected at 0, 1, 4, 8, 12, 24, 36, 48, and 72 hr after inoculation, respectively. Cotton *UBQ7* was used as an internal control. qRT-PCR, quantitative reverse transcription-polymerase chain reaction.

To investigate the tissue-specific expression pattern of *GbNAC1*, qRT-PCR was performed in two cultivars, Xinhai 15 and Xinhai 16. The results indicated that *GbNAC1* was differentially expressed in different organs (Figure 4A, B). Expression was high in euphyllas of both cultivars. However, the expression level of *GbNAC1* was high in cotyledons of Xinhai 15, but low in Xinhai 16. It was identically expressed in roots, stems, and stem apices in both cultivars.

After inoculation of Xinhai 16 with *V. dahliae*, the expression of *GbNAC1* in leaves of control and treated plants differed over time. The expression level decreased in infected plants compared to control from 1–48 hr, but increased at 72 hr (Figure 4C).

### Promoter analysis of GbNAC1 in transgenic Arabidopsis

**Figure 5:**
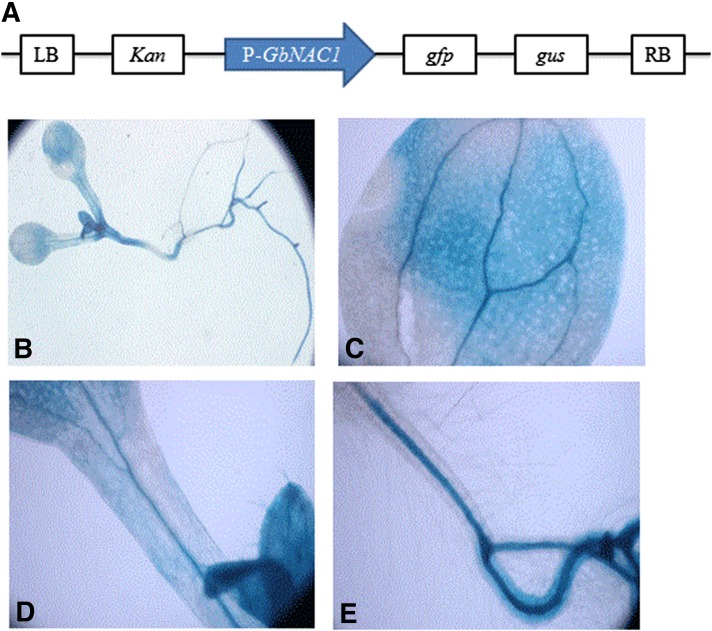
Histochemical GUS staining of different tissues in pGbNAC1:: GUS *Arabidopsis*. (A) Schematic diagram of pGbNAC1:: GUS. Drawing is not to scale. (B) Staining of whole transgenic plant. Staining of leaf (C), stem (D), and root (E) tissues.

To elucidate the spatial expression of *GbNAC1*, GUS activity was analyzed under the control of the *GbNAC1* promoter. Histochemical staining in transgenic *Arabidopsis* seedlings showed that *GbNAC1* expression was present in leaves, stems, and roots (Figure 5A), and was particularly high in vascular bundles (Figure 5, B–D).

### Silencing of GbNAC1 reduced resistance to V. dahliae

**Figure 6 V.**
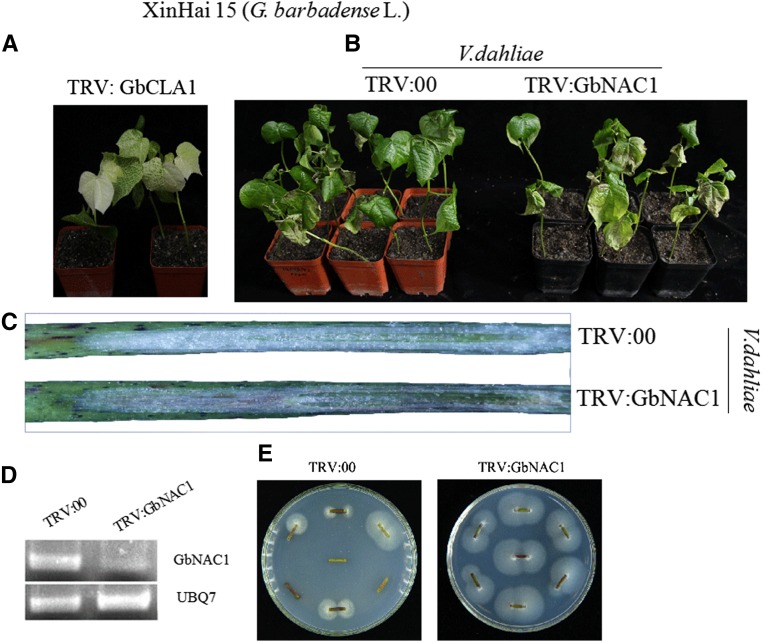
*dahliae*-resistant analysis of *GbNAC1*-silencing (TRV: GbNAC1) and control (TRV: 00) in Xinhai15 (*G. barbadense* L.). (A) The albino phenotype of true leaves after 10 d with the VIGS method. (B) The disease phenotype of TRV: 00 plants and TRV: GbNAC1 plants by inoculation with *V. dahliae* after 10 d. (C) Diagonal plane of stem by inoculation with *V. dahliae* after 10 d. (D) RT-PCR analysis of the expression of *GbNAC1* in TRV: GbNAC1 and TRV: 00. Cotton gene *UBQ7* was used as an internal control. (E) The fungal renewal cultivation of *V. dahliae* inoculation stem sections on PDA medium. (F) Disease index after inoculation with *V. dahliae*. PDA, potato dextrose agar; RT-PCR, reverse transcription-polymerase chain reaction; VIGS, virus-induced gene silencing.

To explore the function of *GbNAC1* in cotton plant defense against *V. dahliae*, VIGS was used to silence the gene expression of *GbNAC1*. The *GbCla1* gene was used as a visual marker for VIGS to monitor efficiency and reliability. The true leaves displayed an albino phenotype after cotyledons were hand-infiltrated by *Agrobacterium* carrying TRV:GhCla1 (Figure 6A). Compared to the expression of *GbNAC1* in TRV:00 controls, expression in TRV:GbNAC1 plants was suppressed after 2 wk (Figure 6D). After inoculation with *V. dahliae* strain V991, typical disease symptoms were seen in TRV:00 plants and TRV:GbNAC1 plants after 10 d; however, disease symptoms, including leaf chlorosis and necrosis, were more severe in TRV:GbNAC1 plants than in TRV:00 plants (Figure 6B and Supplemental Material, Figure S1). In addition, if a plant is infected by *V. dahliae*, xylem vessels turn brown. The diagonal plane of the stem was more apparent in TRV:GbNAC1 plants than in TRV:00 plants (Figure 6C), and the fungal renewal cultivation showed that the disease conditions of TRV:GbNAC1 plants were more serious compared with controls (Figure 6E). The increase in the disease index indicated that downregulation of *GbNAC1* via VIGS reduced *V. dahliae* resistance in cotton (Figure 6F).

### Overexpression of GbNAC1 enhanced resistance against V. dahliae infection in Arabidopsis

**Figure 7:**
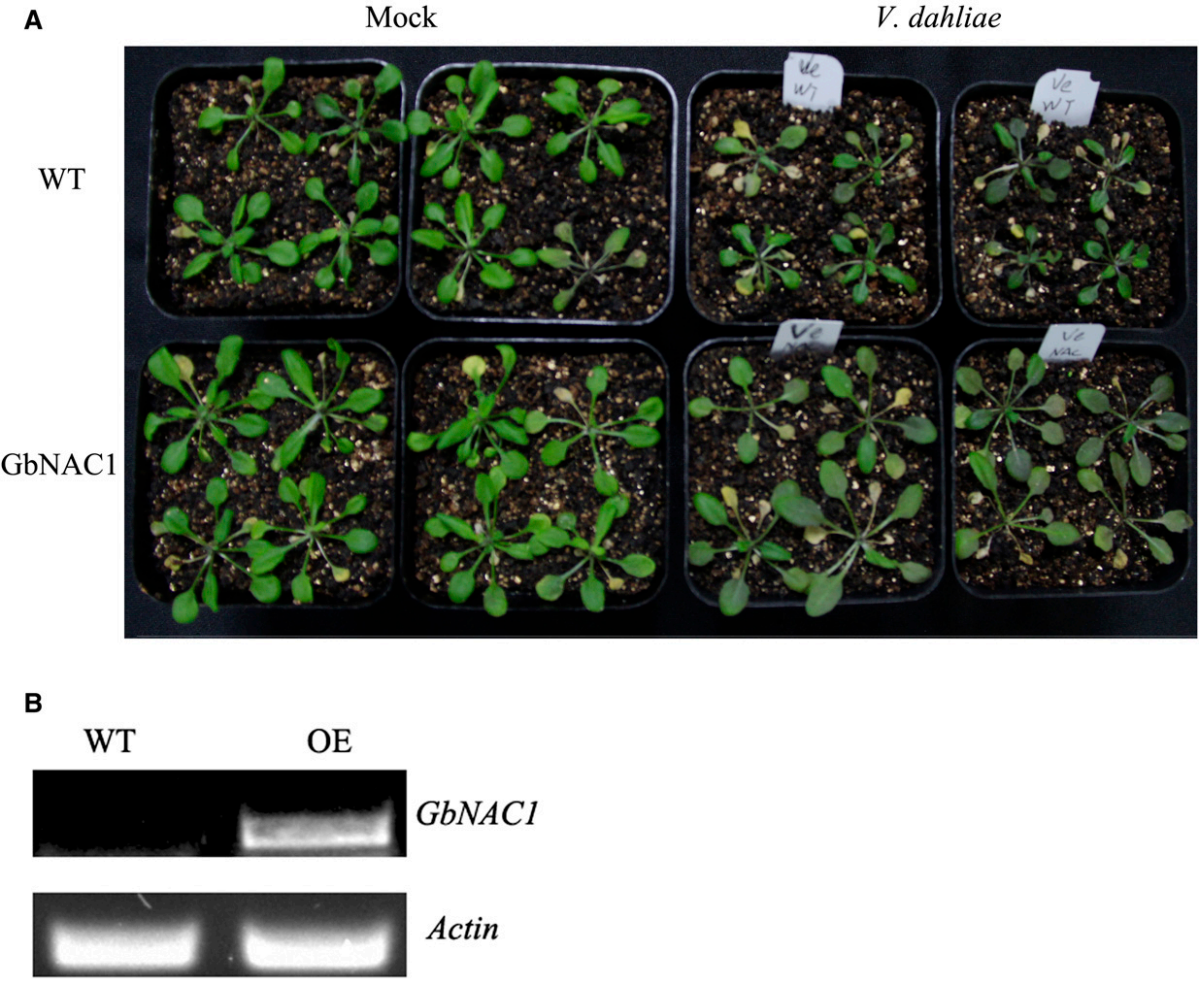
Disease-resistant analysis of overexpressing transgenic *Arabidopsis*. (A) The resistance of *GbNAC1* to *V. dahliae* in overexpressed *Arabidopsis*. (B) Semiquantity expression of *GbNAC1* in wild-type (WT) and overexpression transgenic plants. *Arabidopsis* gene *Actin* was used as an internal control.

To further determine the functions of *GbNAC1*, the phenotype of the T3 generation of overexpressing transgenic *Arabidopsis* plants was observed compared to wild-type plants. According to the semiquantity, the *GbNAC1* was overexpressed (Figure 7B). Overexpressing plants confirmed the function of *GbNAC1* in resistance against *V. dahliae*. During *V. dahliae* infection, wild-type plants and transgenic plants displayed different phenotypes. Wild-type plants were susceptible to *V. dahliae*; however, *GbNAC1* transgenic plants exhibited enhanced resistance against infection, with less chlorosis and necrosis after 10 d (Figure 7A).

### Phenotype of transgenic Arabidopsis and response to abiotic stress

**Figure 8:**
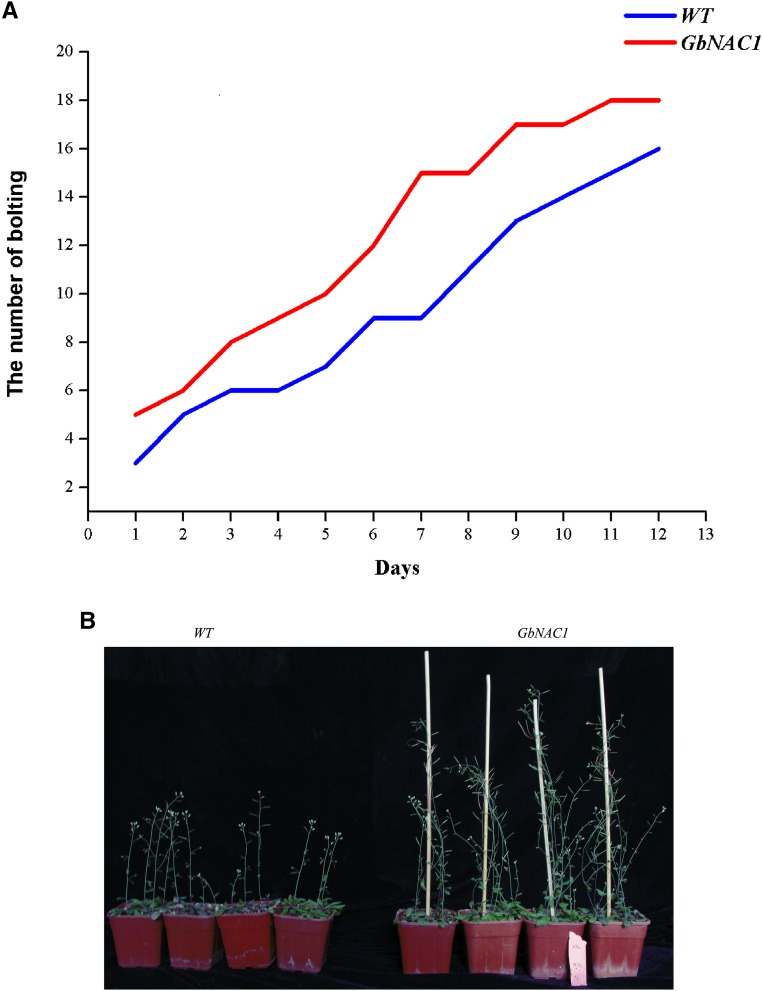
Phenotype analysis of overexpressing transgenic *Arabidopsis*. (A) Statistical analysis of the amount of bolting in wild-type (WT) and transgenic plants. (B) Phenotype of wild-type and overexpression transgenic mature plants.

Compared with wild-type plants, transgenic plants grew quickly in the seedling stage (Figure 8A) and overexpressing plants reach the bolting stage earlier. In addition, transgenic *Arabidopsis* plants were taller when mature (Figure 8B). The results indicate that *GbNAC1* is involved in plant growth and development.

**Figure 9:**
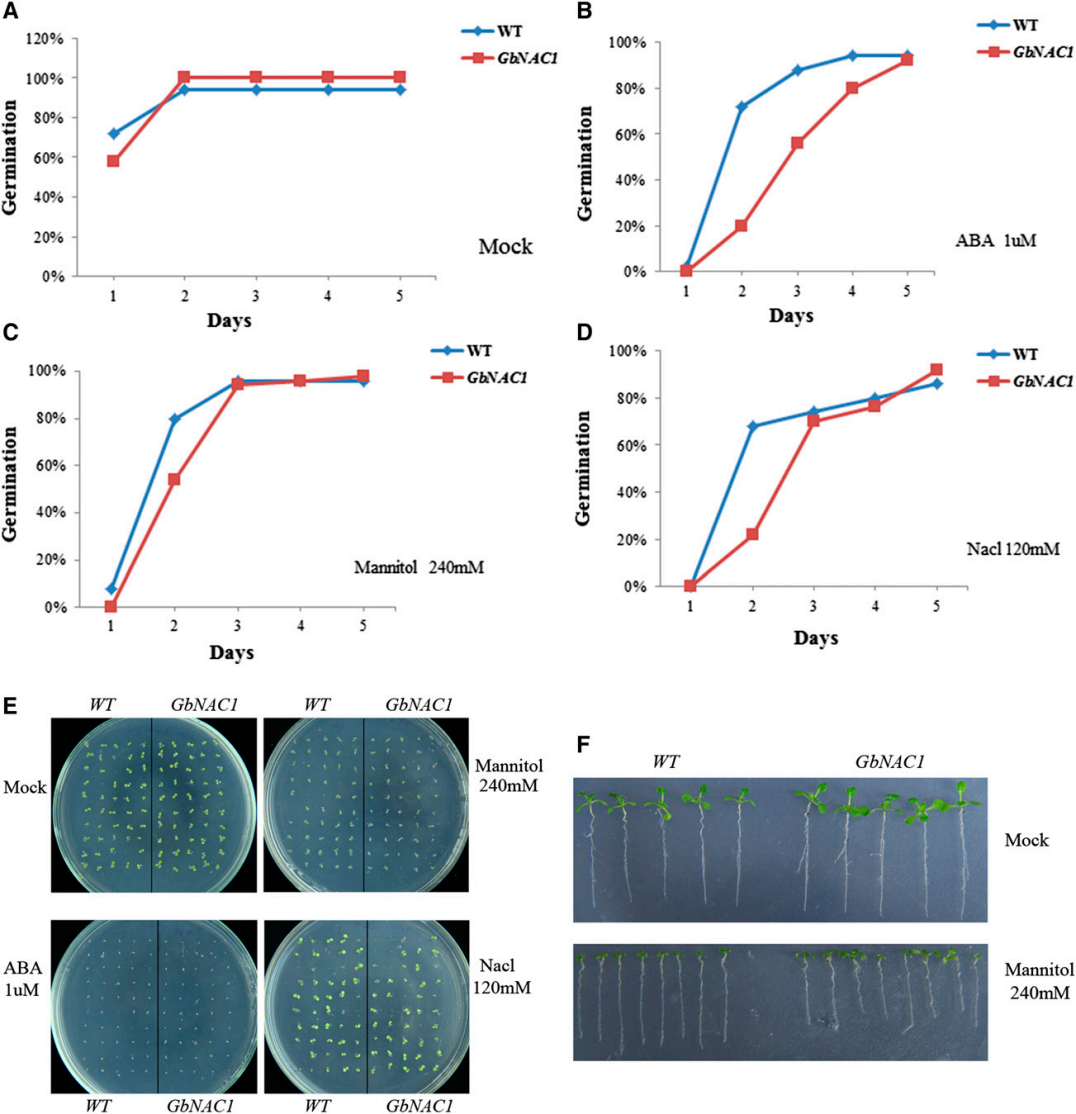
Analysis of transgenic *Arabidopsis* under stress conditions. Seed germination of wild-type and transgenic plants on (A) blank MS or MS-containing (B) ABA (1 μM), (C) mannitol (240 mM), and (D) NaCl (120 mM), respectively. (E) Germination condition of wild-type and transgenic on plants MS containing blank, ABA, Mannitol, and NaCl after 5 d. (F) Phenotype of wild-type and transgenic plant seedlings on MS (blank and Mannitol, respectively). ABA, abscisic acid; MS, mannitol salt; WT, wild-type.

Different stress treatments revealed differences in germination between overexpressing *Arabidopsis* plants and wild-type. Under normal conditions, the germination rate of transgenic plants was higher than wild-type within the first 5 d (Figure 9A). Under 1 μM ABA, the germination of transgenic plants was delayed and the rate was lower than that of wild-type (Figure 9B). There was no obvious difference in germination between transgenic plants and wild-type in 240 mM mannitol (Figure 9C). In 120 mM NaCl, the germination rate of transgenic plants was lower, and then higher, than that of wild-type (Figure 9D). The germination conditions of transgenic and wild-type plants under different abiotic stresses are displayed in Figure 9E. Wild-type and transgenic seedlings showed different growth characteristics after germination in the mannitol treatment. Transgenic seedling roots were longer than wild-type under normal growth conditions (Figure 9F). Both wild-type and transgenic seedlings were inhibited by osmotic stress, but transgenic plants were more sensitive than wild-type plants (Figure 9F). The results suggested that *GbNAC1* is also involved in the downregulation of abiotic stress responses.

## Discussion

NAC is an important terrestrial plant-specific transcription factor family that is involved in a wide range of regulatory roles in the biotic and abiotic stress response, growth, and development. In recent years, a number of NAC transcription factors have been reported to be involved in biotic stress responses.

Based on the previous transcriptome sequencing of sea-island cotton in response to *V. dahliae* (Sun *et al.* 2013b), we identified the differential expression of a unigene fragment (designated as *GbNAC1*). *GbNAC1* encodes a NAC protein with 180 amino acids containing three NAC substructure domains (C–E) (Figure 1A and Figure 2). *GbNAC1* lacks the A and B substructure domains, which might, to some extent, influence the function of *GbNAC1*, though the potential influence of this lack of domains is unknown. According to a previous study, substructure domains C and D contain nuclear localization signals (Olsen *et al.* 2005); thus, *GbNAC1* was localized in the nucleus of transgenic tobacco epidermal cells (Figure 3). Furthermore, based on the homologous sequence alignment of TERN NAC-TFs, these NAC proteins have a conserved WKATGSPG sequence, similar to WRKYGQK from WRKY proteins (Figure 1A), which suggests that this conserved domain may function in DNA binding and that the NAC-TF and WRKY TF families may have a common evolutionary ancestor (Yabuki *et al.* 2005; Babu *et al.* 2006).

*GbNAC1* was expressed in roots, stems, and leaves in both Xinhai 15 and Xinhai 16 (two varieties of *G. barbadense* L.), and was especially highly expressed in leaves (Figure 4). The GUS results showed that the expression of *GbNAC1* was focused in the vascular bundle (Figure 5). As reported previously, *V. dahliae* invades from the root system, entering and spreading upward through vessels, damaging those vessels and hindering the transportation of water, thereby inducing the disease symptoms (Sal’kova and Guseva 1965). During *V. dahliae* infection, *GbNAC1* expression initially decreased, then increased at 72 hr, indicating that *GbNAC1* expression in vascular bundles may be involved in the response to *V. dahliae*.

Overexpression of *NTL6*, an active NAC protein, increased resistance to *Pseudomonas syringae* in transgenic *Arabidopsis* (Seo *et al.* 2010). *SISRN1*, a tomato NAC gene, was involved in the response to *Botrytis cinerea* (Liu *et al.* 2014a). *NTP1* and *NTP2*, two NAC proteins from potato and *N. benthamiana*, are involved in the response to *P. infestans* (McLellan *et al.* 2013). In this study, the function of *GbNAC1* in resistance to *V. dahliae* was examined using VIGS and overexpression in *Arabidopsis*. The VIGS result showed that resistance to *V. dahliae* was reduced when *GbNAC1* was silenced in the resistant variety (Xinhai15). Furthermore, *GbNAC1*-overexpressing *Arabidopsis* plants showed enhanced resistance to Verticillium wilt compared to wild-type plants. This demonstrates that *GbNAC1* plays a positive regulatory role in resistance to *V. dahliae*. This study serves as a foundation for the further functional study of NAC genes in cotton. Overexpression of *OsNAC9* could enhance drought resistance under the control of the *RCc3* promoter in rice (Redillas *et al.* 2012). Not all NAC proteins were positive regulators of abiotic stress; for example, *ATAF1* played a negative role in drought stress (Lu *et al.* 2007).

In this study, *GbNAC1*-overexpressing *Arabidopsis* plants showed more vigorous growth both as seedlings and mature plants, especially in terms of bolting, than wild-type plants. However, in the presence of abiotic stressors, such as NaCl, ABA, and mannitol, wild-type plants fared better than overexpressing plants in terms of seed germination and seedling growth. These results suggest that *GbNAC1* is involved in plant growth and development and also responds to biotic and abiotic stress, though the function of NAC genes in cotton requires further study.

## Supplementary Material

Supplemental Material
