# Supplementary material for: Characterization, Expression, and Functional Analysis of a Novel NAC Gene Associated with Resistance to Verticillium Wilt and Abiotic Stress in Cotton
Source: G3 (Bethesda). 2016 Oct 24;6(12):3951–61. doi: 10.1534/g3.116.034512 (PMC5144965; doi:10.1534/g3.116.034512)
Supplement: Supplemental Material [file supp_6_12_3951__index.html]

Characterisation, Expression, and Functional Analysis of a Novel NAC Gene Associated with Resistance to Verticillium Wilt and Abiotic Stress in Cotton — Characterization, Expression, and Functional Analysis of a Novel NAC Gene Associated with Resistance to Verticillium Wilt and Abiotic Stress in Cotton — Supplemental Material 

# Characterization, Expression, and Functional Analysis of a Novel NAC Gene Associated with Resistance to Verticillium Wilt and Abiotic Stress in Cotton

## Supplemental Material for Wang *et al.*, 2016

**Files in this Data Supplement:**

- Figure S1 - *V. dahlia*-resistant analysis of *GbNAC1*-silencing (TRV: GbNAC1) and control (TRV: 00) in Xinhai15 (*G. barbadense* L.). (.pdf, 37 KB)
