# Supplementary material for: Characterization, Expression, and Functional Analysis of a Novel NAC Gene Associated with Resistance to Verticillium Wilt and Abiotic Stress in Cotton
Source: G3 (Bethesda). 2016 Oct 24;6(12):3951–61. doi: 10.1534/g3.116.034512 (PMC5144965; doi:10.1534/g3.116.034512)
Supplement: Supplemental Material [file supp_g3.116.034512_FigureS1.pdf]

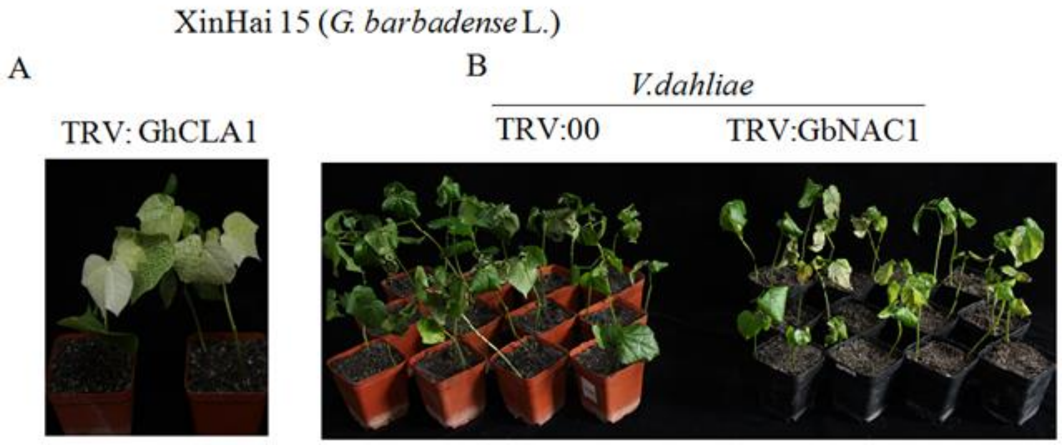

Figure S1. *V. dahliae*-resistant analysis of *GbNAC1*-silencing (TRV: GbNAC1) and control (TRV: 00) in Xinhai15 (*G. barbadense* L.). (A) The albino phenotype of true leaves after 10 days with the method of VIGS. (B) The disease phenotype of TRV: 00 plants and TRV: GbNAC1 plants by inoculation with *V. dahliae* after 10 days.
